# Inhibition of Hedgehog Signaling Decreases Proliferation and Clonogenicity of Human Mesenchymal Stem Cells

**DOI:** 10.1371/journal.pone.0016798

**Published:** 2011-02-03

**Authors:** Magali Plaisant, Sophie Giorgetti-Peraldi, Marike Gabrielson, Agnes Loubat, Christian Dani, Pascal Peraldi

**Affiliations:** 1 CNRS UMR6543, Institute of Biology, Development and Cancer, Faculté de Médecine, Nice, France; 2 Université de Nice Sophia Antipolis, IFR50, Faculté de Médecine, Nice, France; 3 INSERM U895, Team Cellular and Molecular Physiopathology of obesity and diabetes, Nice, France; Pennington Biomedical Research Center, United States of America

## Abstract

Human mesenchymal stem cells (hMSC) have the ability to differentiate into osteoblasts, adipocytes and chondrocytes. We have previously shown that hMSC were endowed with a basal level of Hedgehog signaling that decreased after differentiation of these cells. Since hMSC differentiation is associated with growth-arrest we investigated the function of Hh signaling on cell proliferation. Here, we show that inhibition of Hh signaling, using the classical inhibitor cyclopamine, or a siRNA directed against Gli-2, leads to a decrease in hMSC proliferation. This phenomenon is not linked to apoptosis but to a block of the cells in the G0/G1 phases of the cell cycle. At the molecular level, it is associated with an increase in the active form of pRB, and a decrease in cyclin A expression and MAP kinase phosphorylation. Inhibition of Hh signaling is also associated with a decrease in the ability of the cells to form clones. By contrast, inhibition of Hh signaling during hMSC proliferation does not affect their ability to differentiate. This study demonstrates that hMSC are endowed with a basal Hedgehog signaling activity that is necessary for efficient proliferation and clonogenicity of hMSC. This observation unravels an unexpected new function for Hedgehog signaling in the regulation of human mesenchymal stem cells and highlights the critical function of this morphogen in hMSC biology.

## Introduction

Mesenchymal stem cells (MSC) reside in a variety of tissues and can differentiate into adipocytes, osteoblasts, and chondrocytes. The number of MSC present in the organism is dependent upon their rate of differentiation and self-renewal properties, i.e. their ability to proliferate without losing their differentiation properties. Both processes are highly regulated and their deregulation alters the homeostasis of various organs and can give rise to pathologies such as obesity and osteoporosis [1], [2].

The molecular mechanisms presiding upon hMSC self-renewal are not completely unraveled. hMSC self-renewal depends upon FGF [3], Activin A [4] and Wnt [5], but other factors are likely to be involved. Hedgehog, originally discovered in Drosophila, is an important morphogen that controls a variety of mammalian developmental phenomena such as induction of the ventral cell fates in the central nervous system and patterning of the anterior-posterior axis of the developing limb [6], [7], [8], [9]. Hh has also homeostatic functions in post embryonic tissues controlling cell growth, axon guidance and cell differentiation. Shh controls self-renewal of neural stem cells [10], [11], [12], hair follicle stem cells [13] and mammary stem cells [14]. Moreover, an abnormal activity of Hh signaling is thought to be responsible for the anarchic self-renewal properties of several cancer stem cells such as the one found in gastrointestinal, breast and brain cancers [9], [15], [16].

We have shown that Hh controls the differentiation properties of hMSC, through a species-specific effect [17], [18]. Moreover, differentiation of hMSC into adipocytes [17] or osteoblasts [18] is associated with a decrease in Hh signaling. Since hMSC differentiation is associated with growth-arrest, Hh signaling appeared as a candidate for controlling proliferation of these cells. As we previously shown that stimulation of Hh signaling did not affect the proliferation of hMSC we investigated the effect of an inhibition of Hh signaling on hMSC proliferation.

Schematically, Hh signaling is initiated by the binding of Hh (Sonic Hedgehog, Indian Hedgehog or Desert Hedgehog), to its receptor, Patched (Ptc). Upon binding, Ptc relieves its suppression on Smo [15]. Smo is then localized into the primary cilium of the cell, an organelle playing a critical role in Hh signaling [19], [20]. There, Smo activates an intracellular cascade that results in the stabilization of Gli2. This transcription factor translocates into the nucleus and induces the transcription of Hh target genes, such as Gli1, a reliable marker of Hh signaling [7], [9], [21]. Gli2 is a critical component of Hh signaling and its inactivation leads to an inhibition of Hh signaling.

Here, we show for the first time that inhibition of Hh signaling inhibits the proliferative and clonogenic properties of hMSC, without modifying their ability to differentiate.

## Results

### Hh signaling is modulated during cell differentiation

Previous studies indicated that differentiation of hMSC into adipocytes and osteoblasts is associated with a decrease in Hh signaling [17], [18]. Since hMSC differentiation is associated with cell growth-arrest we investigated the function of Hh signaling in hMSC proliferation. To this purpose, we used hMADS cells which are human adipose-derived MSC. These cells are multipotent, display a normal karyotype and are not transformed [22].

**Figure 1:**
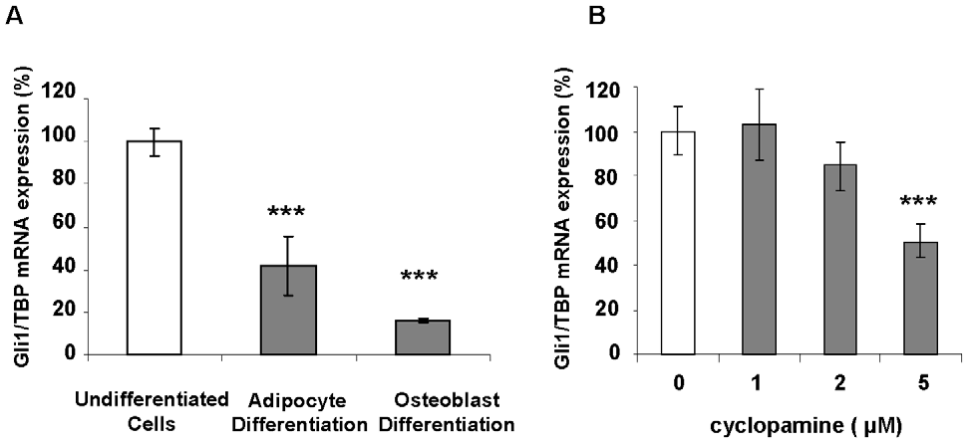
Hedgehog signaling is downregulated during differentiation of hMADS cells. hMADS cells were kept undifferentiated or differentiated into adipocytes or osteoblasts (**A**), or treated with increasing concentrations of cyclopamine for 7 days (**B**). Gli-1 expression was evaluated by real-time quantitative reverse transcription-polymerase chain reaction (RT-PCR). Results are representative of three independent experiments.

We have previously shown that activation of Hh signaling by purmorphamine did not modify proliferation of hMSC [17]. However, Hh signaling was found to be active in undifferentiated cells and decreased after cell differentiation. This is illustrated in Figure 1 where hMADS were differentiated into adipocytes, osteoblasts or kept in an undifferentiated state. Gli-1 mRNA expression, which has been established to reflect Hh signaling activity [7], [9], [21], was monitored by real time RT-PCR. As observed, hMADS differentiation into osteoblasts or adipocytes is associated with a decrease in Hh signaling.

In order to investigate the effect of the inhibition of Hh signaling on hMSC self-renewal we used the classical inhibitor cyclopamine [23]. This molecule, which binds directly to smoothened, is a classical specific inhibitor of the Hh pathway that has been extensively used in *in vivo* and *in vitro* experiments. As observed (Figure 1B) cyclopamine induces a 50% decrease in Gli-1 expression at 5 µM. It was the maximal concentration we used in our subsequent experiments since its effect on Hh signaling was similar to the one observed after cell differentiation, and to avoid possible specificity issues [24].

### Inhibition of Hh signaling decreases hMADS cell proliferation

**Figure 2:**
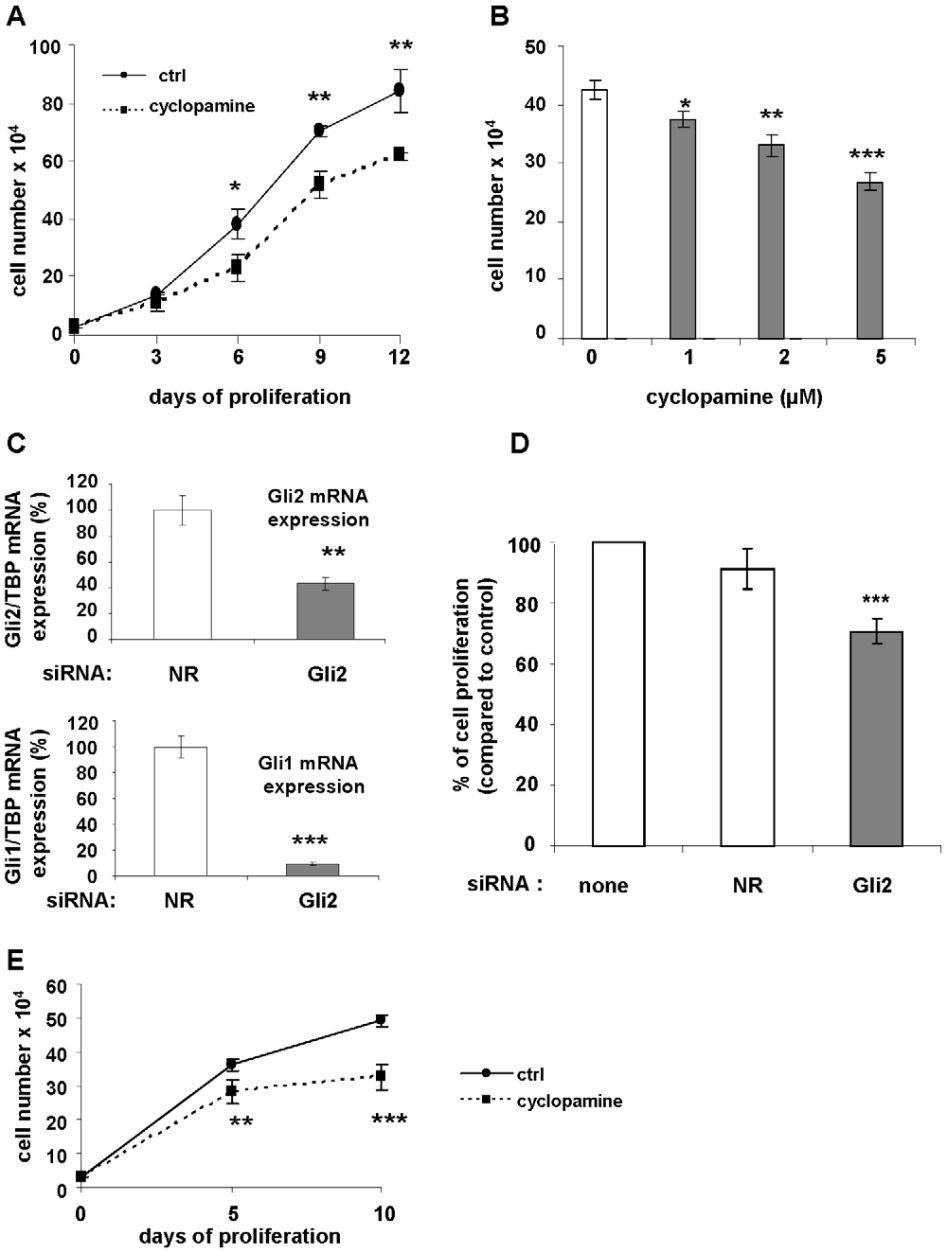
Inhibition of Hh signaling decreases hMSC proliferation. (**A**): hMADS cells were grown in presence of FGF-2 (2.5 ng/ml) and treated every other day with (square) or without (circle) cyclopamine (5 µM) for 12 days, then counted at the indicated time. (**B**): Dose response of cyclopamine on cell proliferation. Cells were grown for 6 days with increasing doses of cyclopamine and counted. (**C**): Cells were transfected with a non relevant (NR) siRNA or a siRNA directed against Gli 2. After 72 hours mRNA were extracted and Gli2 and Gli1 expressions were evaluated by quantitative RT-PCR. (**D**): Number of hMADS cells 3 days after transfection with a non relevant siRNA or a Gli2 siRNA. The data presented are means ± SE of three independent experiments performed in triplicate. (**E**): Proliferation curve of hMSC from bone marrow. Cells were treated with cyclopamine (5 µM) and counted after 5 and 10 days. Results are representative of three independent experiments.

We tested the effect of an inhibition of Hedgehog signaling on cell proliferation. First, cells were treated or not with cyclopamine (5 µM) and counted every 3 days (Figure 2A). As observed, cyclopamine induced a 25% decrease in cell proliferation after 12 days. A dose-response experiment was performed (Figure 2B). 1 µM cyclopamine induced a modest decrease in cell proliferation. This inhibition reaches 37% at 5 µM. Similar results were obtained with another hMADS cell line (data not shown).

To make sure that the inhibition of proliferation observed with cyclopamine was specific of a decrease of Hh signaling, we used a siRNA directed against Gli-2. Indeed, Gli-2 is a critical transcription factor in the Hh signaling pathway and its activation is necessary for Gli-1 expression [9], [15]. Cells were transfected with a non relevant (NR) siRNA or a siRNA directed against Gli-2 (Figure 2C), RNAs were extracted and Gli-2 and Gli-1 mRNAs expression monitored using quantitative RT-PCR. As observed, Gli-2 siRNA induces a 60% decrease in Gli-2 expression. As expected, this was accompanied by a 90% inhibition of Gli-1 expression.

We then tested the effect of Gli-2 silencing on hMADS proliferation. hMADS were transfected or not with a non-relevant siRNA or a siRNA directed against Gli-2. After 5 days cells were counted (Figure 2D). Inhibition of Gli-2 expression leads to a 27% decrease in cell proliferation. Since cyclopamine and siRNA against Gli-2 provided similar results all subsequent experiments were performed using cyclopamine.

We tested the effect of cyclopamine on the proliferation of hMSC primary cultures obtained from bone marrow. Cells were treated with cyclopamine and counted after 5 and 10 days (Figure 2E). As observed, cyclopamine decreases bone marrow hMSC cell proliferation, indicating that the effect observed was not restricted to hMADS cells.

### Inhibition of Hh signaling does not affect cell viability

We investigated the mechanism by which Hh inhibition decreases cell proliferation. Hh inhibition has been shown to induce apoptosis in some cell models such as glioblastoma, pancreatic cancer cells and during early embryonic stem cell neurogenesis [25], [26], [27]. We tested the effect of cyclopamine on hMSC viability by performing an annexinV-FITC/propidium iodide (PI) assay. AnnexinV–FITC detects phosphatidylserine at the surface of the cell membrane, an early step of the apoptotic program. Cells positive for both annexin V and PI are considered to be apoptotic while cells positive only for PI are necrotic.

**Figure 3:**
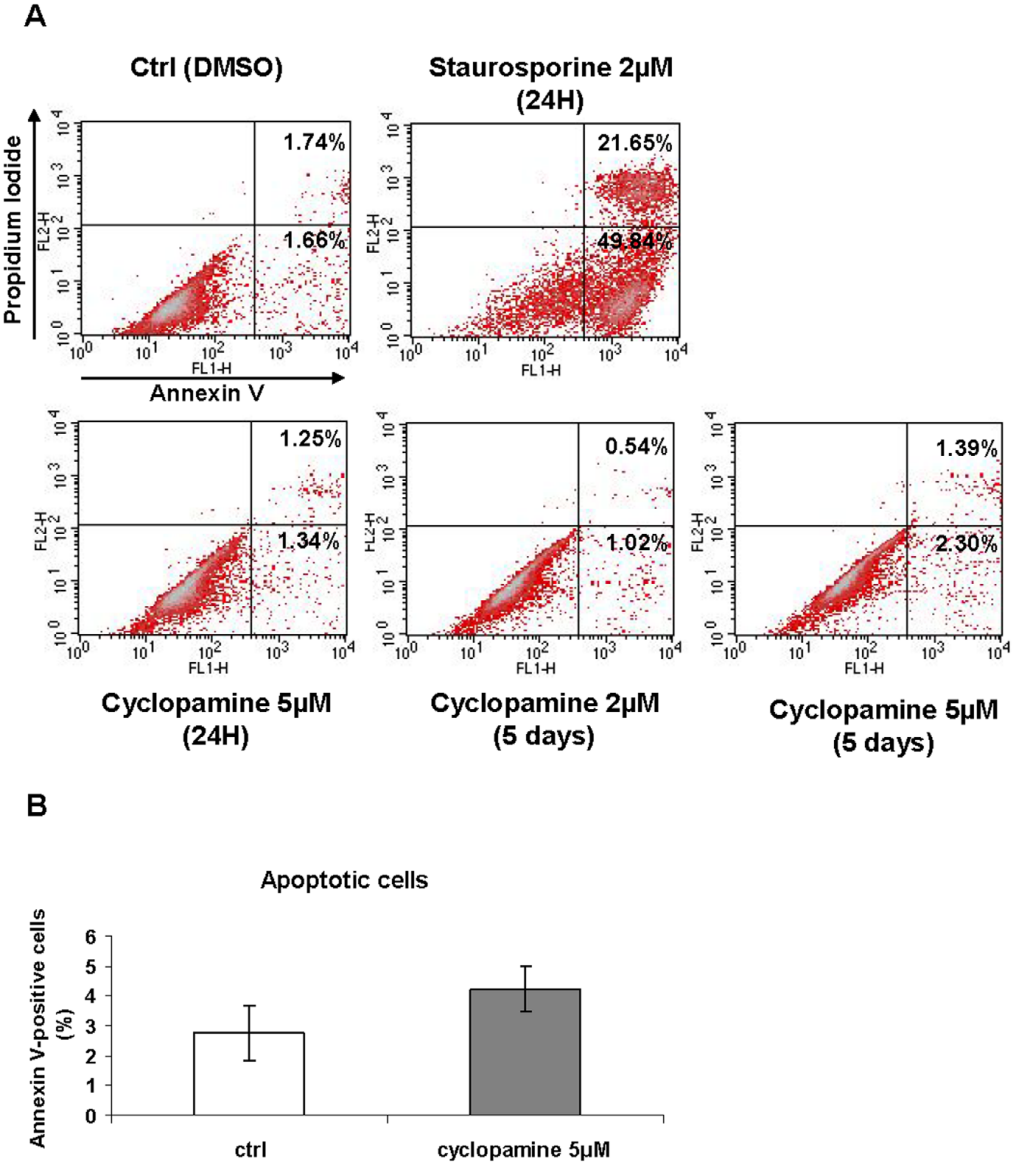
Inhibition of Hh signaling does not affect cell viability. (**A**): Flow cytometry analysis of hMADS cells treated with cyclopamine for 24H or 5 days and labelled with annexin V and propidium iodide (PI). The percentage of cells positively labelled with annexin V (apoptotic cells) is indicated for each condition. Staurosporine was used as a positive control (0.1 µM for 24H). (**B**): Percentage of Annexin V-positive cells after a treatment of 5 days with 5 µM cyclopamine.

hMADS cells were incubated with cyclopamine for 24 hours or 5 days or staurosporine for 24 hours as a positive control for cell death. Staurosporine increased the percentage of positive cells for annexin V (71% of apoptotic cells) (Figure 3A). By contrast, cyclopamine (5 µM) did not affect significantly the amount of annexinV-positive cells (Figures 3A and 3B). These results indicate that apoptosis is not implicated in the inhibition of proliferation induced by cyclopamine.

### Inhibition of Hh signaling affects cell cycle

**Figure 4:**
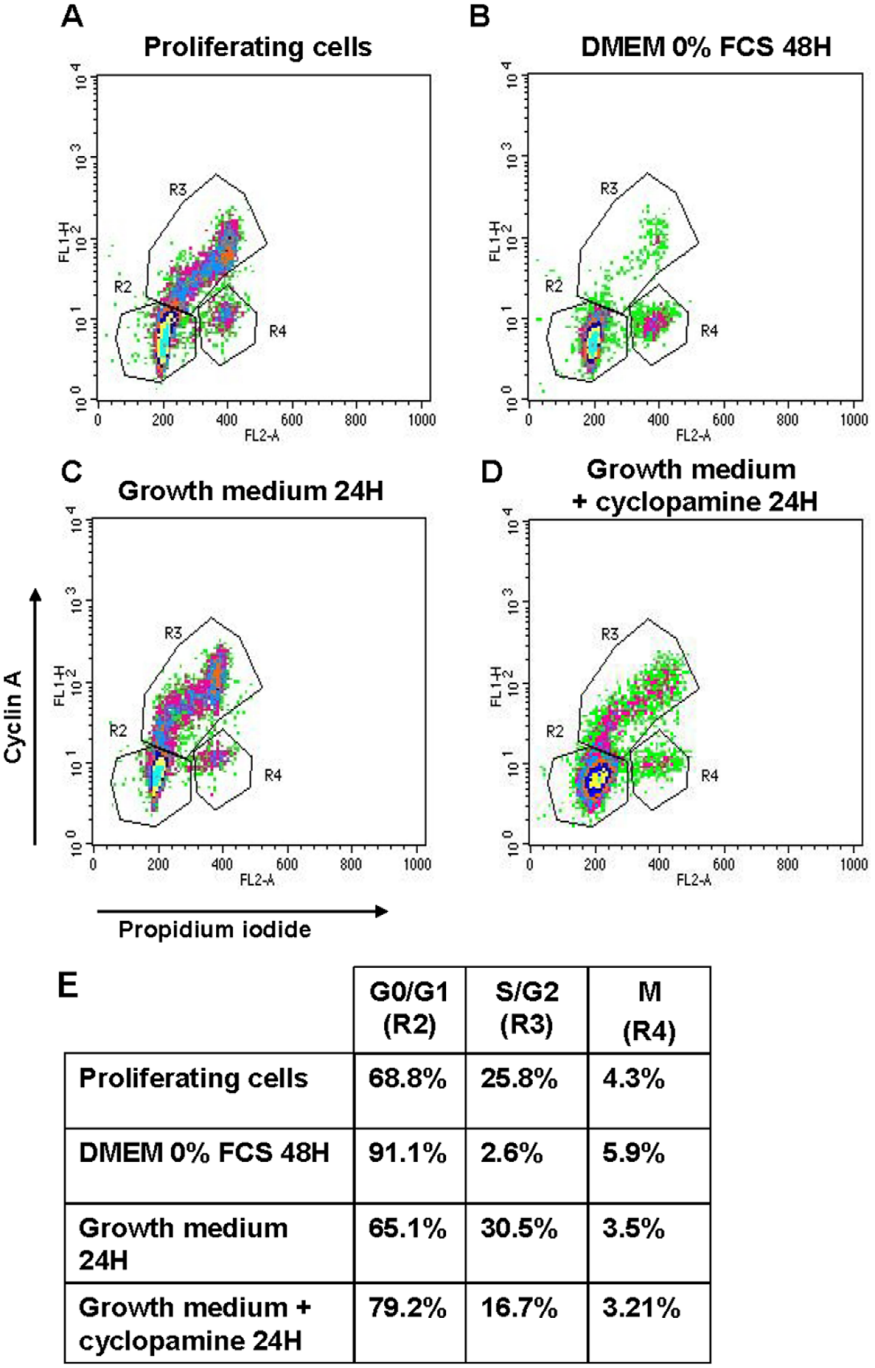
Inhibition of Hh signaling affects hMADS cell cycle. Cell cycle distribution was evaluated by a Cyclin A antibody and PI double labelling and analysed by flow cytometry. (**A**): cell cycle distribution of proliferating hMADS. (**B–D**) hMADS cells were synchronised as described in “Methods” and (**B**) placed in DMEM in absence of serum for 48H or (**C**) incubated in growth medium (10%FCS and FGF-2 (2.5 ng/ml)) for 24H in absence or (**D**) in presence of cyclopamine (5 µM). (**E**) Distribution of cells in G0/G1 (R2 gate), S/G2 (R3 gate) or M (R4 gate) phases of the cell cycle are indicated in the table. Results are representative of three independent experiments.

Since inhibition of Hh signaling does not affect cell viability we investigated whether it affects the cell cycle. Cell cycle distribution was evaluated by cyclin A and PI double labelling and analyzed by flow cytometry (Figure 4). Cyclin A is expressed at the end of the G1 phase/beginning of the S phase through the end of the G2 phase, while PI reflects DNA content of the cells. Thus, cells with low PI and low cyclin A are in G0/G1 phase (gated in R2), cells with high cyclin A and high PI are in S/G2 (R3) while cells with high PI and low cyclin A (R4) are in M phase.

First, cells were synchronized. Incubation of hMADS in DMEM without serum for 48 hours results in 91% of cells arrested in G0/G1 compared to proliferating cells (Figures 4A, 4B and quantification provided in 4E). hMADS cells were then incubated in growth medium (10% FCS and FGF-2) for 24 h in presence or in absence of cyclopamine (5 µM) (Figures 4C, 4D and 4E). As expected, growth medium led to a significant reduction of cells in G0/G1 (65.1% compared to 91.1%) and an increase of cells in S phase. In presence of cyclopamine, the proportion of cells in G0/G1 increased (79.2% compared to 65.1% in absence of cyclopamine) and cells failed to cycle into the S phase (16.7% compared to 30.5% in absence of cyclopamine).

**Figure 5:**
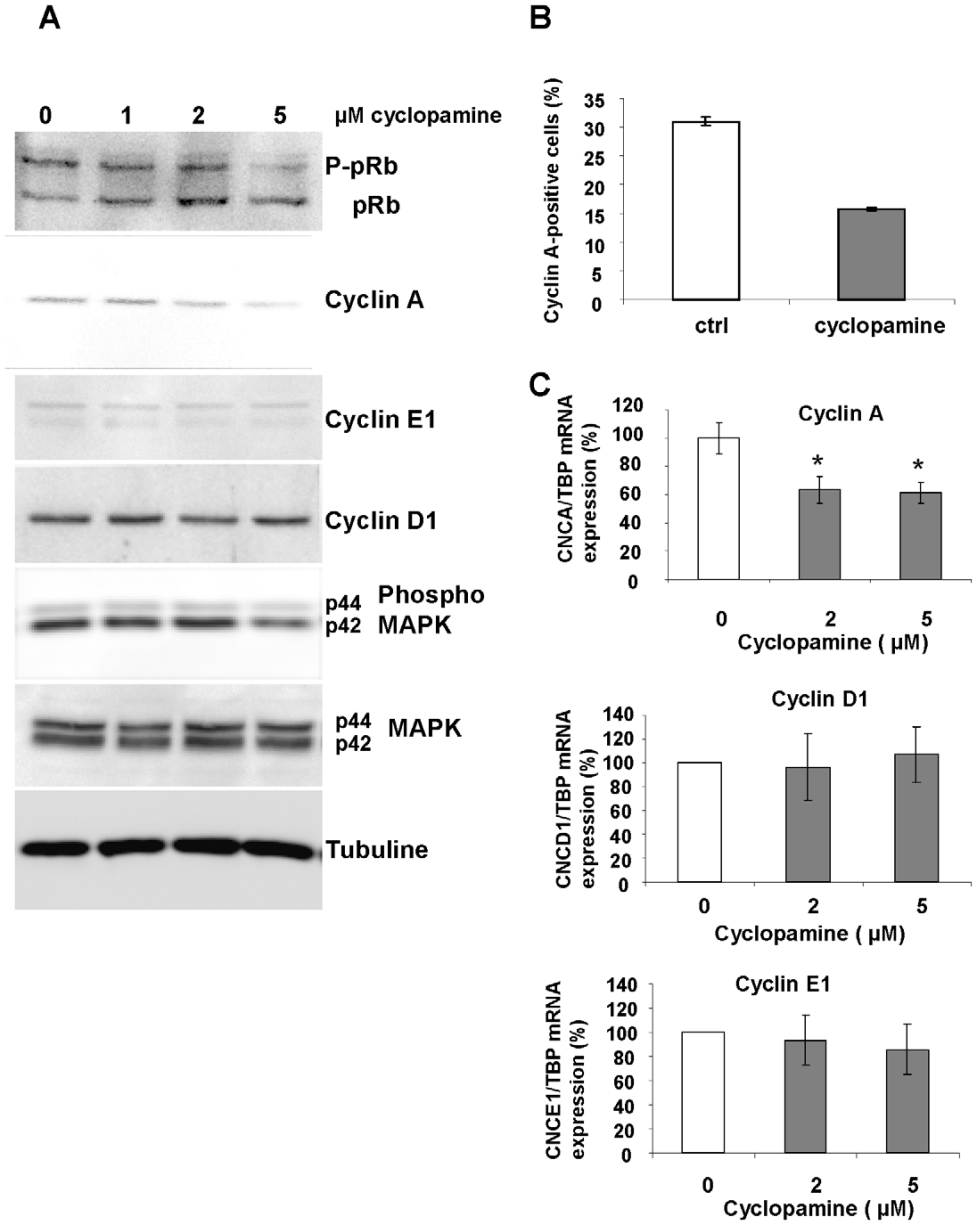
Inhibition of Hh signaling affects key markers of the cell cycle. hMADS cells were synchronised as described in “Methods” and treated with increasing concentrations of cyclopamine for 24H. (**A**): Western blot of pRb (the upper band “P-pRb” is the hyperphosphorylated form of pRb), Cyclins A, E1 and D1, phospho-MAPK and total MAPK. Tubulin antibody was used as a loading control. Western blot are representative of experiments performed at least 3 times. (**B**): Flow cytometry analysis of cyclin A-positive cells after treatment with cyclopamine for 24H (5 µM). (**C**): Quantitative RT-PCR for Cyclins A, D1 and E1. Data are means ± SE of triplicates from an experiment representative of three independent experiments.

Then, we analyzed the expression of several key markers of the cell cycle. Cells were synchronized as described above, and proteins or RNA were extracted. Western Blot experiments indicate that treatment of cells with cyclopamine decreased the hyperphosphorylated form of pRb (P-pRb) while increasing its hypophosphorylated form (Figure 5A). The hypophosphorylated form of the tumor repressor pRB represents its active form that binds E2F to decrease cell proliferation. Cyclopamine induced a (50%+/−8%, p<0.001, measured as described in Methods) decrease in Cyclin A expression (Figure 5A). This was associated with a decrease in the number of Cyclin A positive cells, as measured by FACS (Figure 5B) and a decrease of Cyclin A mRNA expression (Figure 5C). Cyclopamine decreased MAPK phosphorylation by 27% (+/−8%, p<0.01). By contrast, cyclin D1 and Cyclin E1 expressions were not modified significantly (Figure 5A). Q-PCR analysis of cyclin A E1 and D1 were consistent with the results obtained by Western Blot (Figure 5C).

These data indicate that inhibition of Hh signaling delayed the cells in G0/G1 phase and is associated with an increase in the active form of pRB, a decrease in cyclin A expression and of MAP kinase phosphorylation.

### Inhibition of Hh signaling decreases hMADS clonogenicity

**Figure 6:**
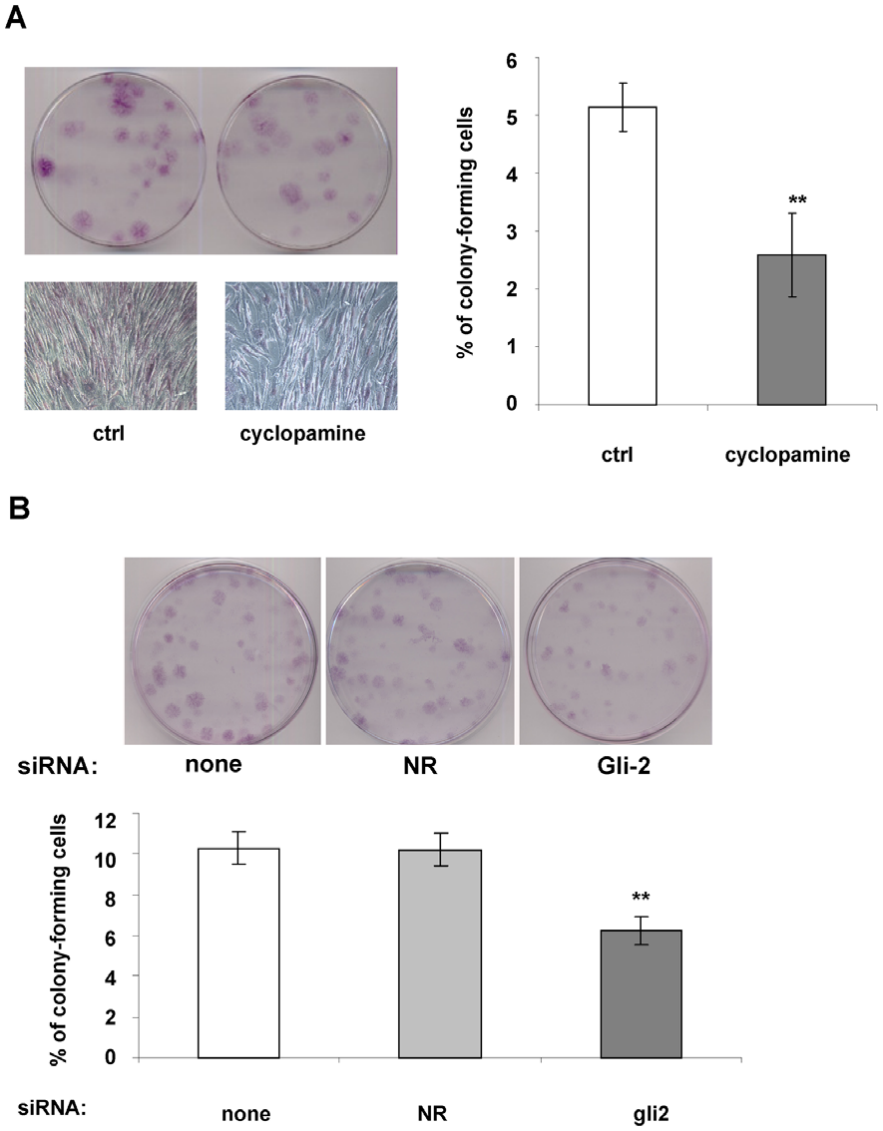
Inhibition of Hh signaling inhibits colony formation of hMADS cells. Cells were plated at clonal density and maintained in medium supplemented with 10% FCS and FGF-2 (2.5 ng/ml) (Ctrl). One day after cell plating, 5 µM cyclopamine was added to the culture medium. (**A**): Photographs of colony formation of hMADS cells 3 weeks after treatment. A magnification of one colony is presented for each condition. (**B**): Photographs of colony formation of hMADS cells transfected with non relevant (NR) siRNA, Gli2 siRNA or only with hyperfect as a control (indicated “none” in the figure). Graphs represent the percentage of colony-forming cells in three independent experiments.

Clonogenicity, i.e. the ability to expand at a single cell level is an important feature of self-renewing stem cells. To test the effect of a decrease Hh signaling on hMSC clonogenicity, cells were plated at low density in presence or in absence of cyclopamine. After 3 weeks, cells were fixed and stained with crystal violet in order to visualize cell colonies (Figure 6A). As observed cyclopamine induces a 50% decrease in the number of colonies. Moreover, in presence of cyclopamine the colonies were less dense than the control colonies, which is consistent with the effect of cyclopamine on cell proliferation.

We tested the specificity of Hh signaling by silencing Gli-2. Cells were transfected with or without a control siRNA or a siRNA against Gli-2. 24 hours later, cells were trypsinised and seeded at low density. After 3 weeks, cells were stained with crystal violet and the colonies were counted. As observed, control siRNA did not affect hMSC clonogenicity but Gli-2 silencing was associated with a 40% decrease in the number of clones (Figure 6B).

### Inhibition of Hh signaling during proliferation of hMSC does not affect their ability to differentiate

**Figure 7:**
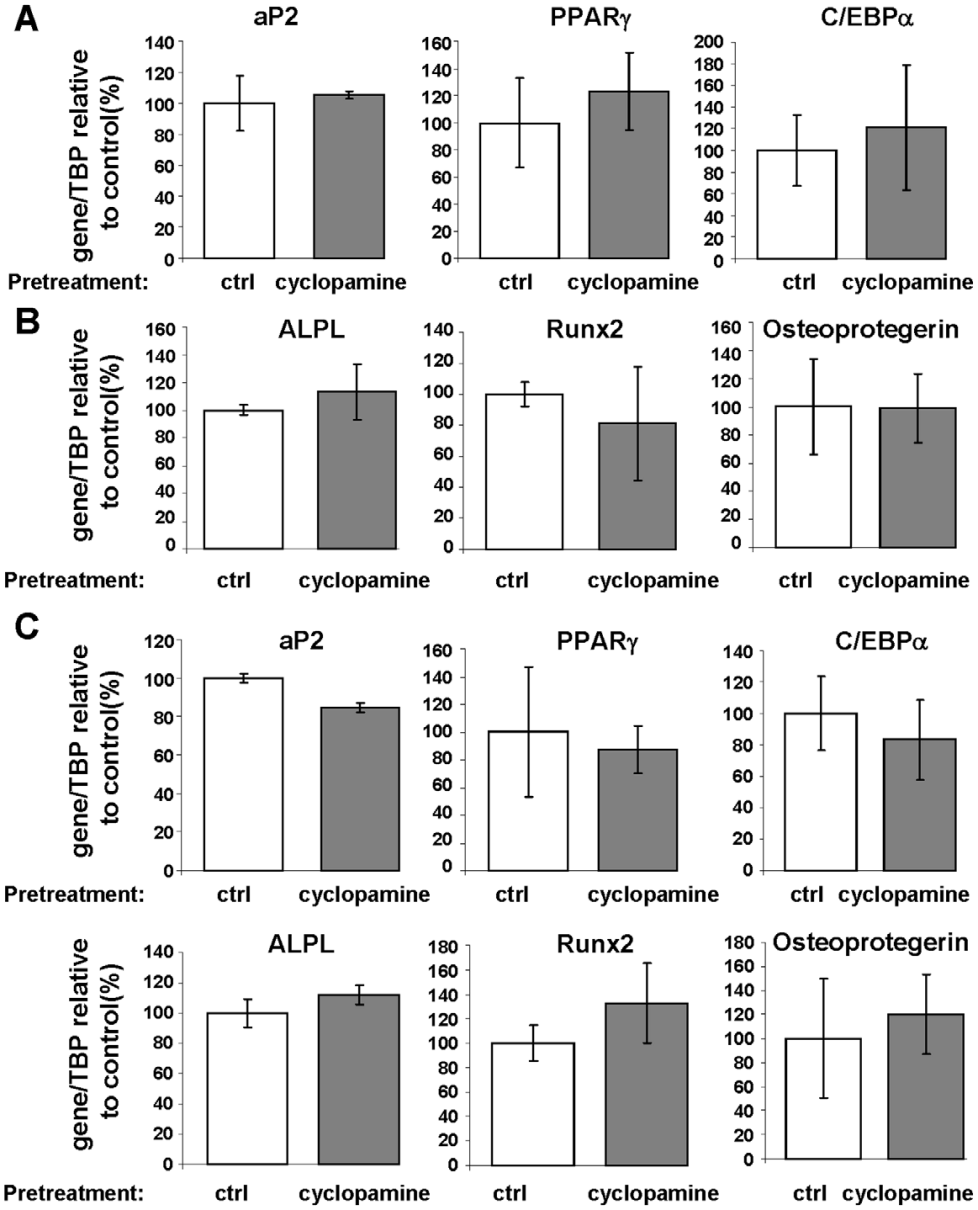
Inhibition of Hh signaling during hMADS proliferation does not affect differentiation potential. hMADS cells were grown in presence or in absence of cyclopamine (5 µM) until confluence for 10 days. Then cyclopamine was removed and cells were induced to differentiate into (**A**) adipocytes or (**B**) osteoblasts or (**C**) both (50% adipogenic and 50% osteogenic media). After 10 days mRNA were extracted and aP2, PPARγ, C/EBP-α, Runx-2, osteoprotegerin and ALPL expressions were evaluated by quantitative RT-PCR.

The effect of cyclopamine on hMSC differentiation has already been reported [17]. Here, we investigate if the inhibition of Hh signaling during cell proliferation affected their ability to differentiate in absence of Hh inhibition. To do this, cells were grown in presence or absence of cyclopamine (5 µM). When cells reached confluence (usually three days later for the cyclopamine-treated cells), cyclopamine was removed from the medium and cells were treated with an adipogenic or an osteogenic medium. After 10 days, RNA were extracted and the expression of adipogenic: aP2, PPARγ and C/EBP-α or osteoblastic markers; Alkaline Phosphatase (ALPL), Runx-2 and osteoprotegerin was evaluated by quantitative PCR. No significant difference was observed between conditions (Figures 7A and 7B).

We then analyzed if a pre-treatment with cyclopamine could modify the ability of the cells to differentiate preferentially into adipocytes or osteoblasts. To do this, after cyclopamine treatment, cells were treated with a “dual” differentiation medium allowing the differentiation into adipocytes and osteoblasts. Expression of differentiation markers was then analyzed. As observed in Figure 7C pre-treatment with cyclopamine did not favor one type of differentiation over the other.

**Figure 8:**
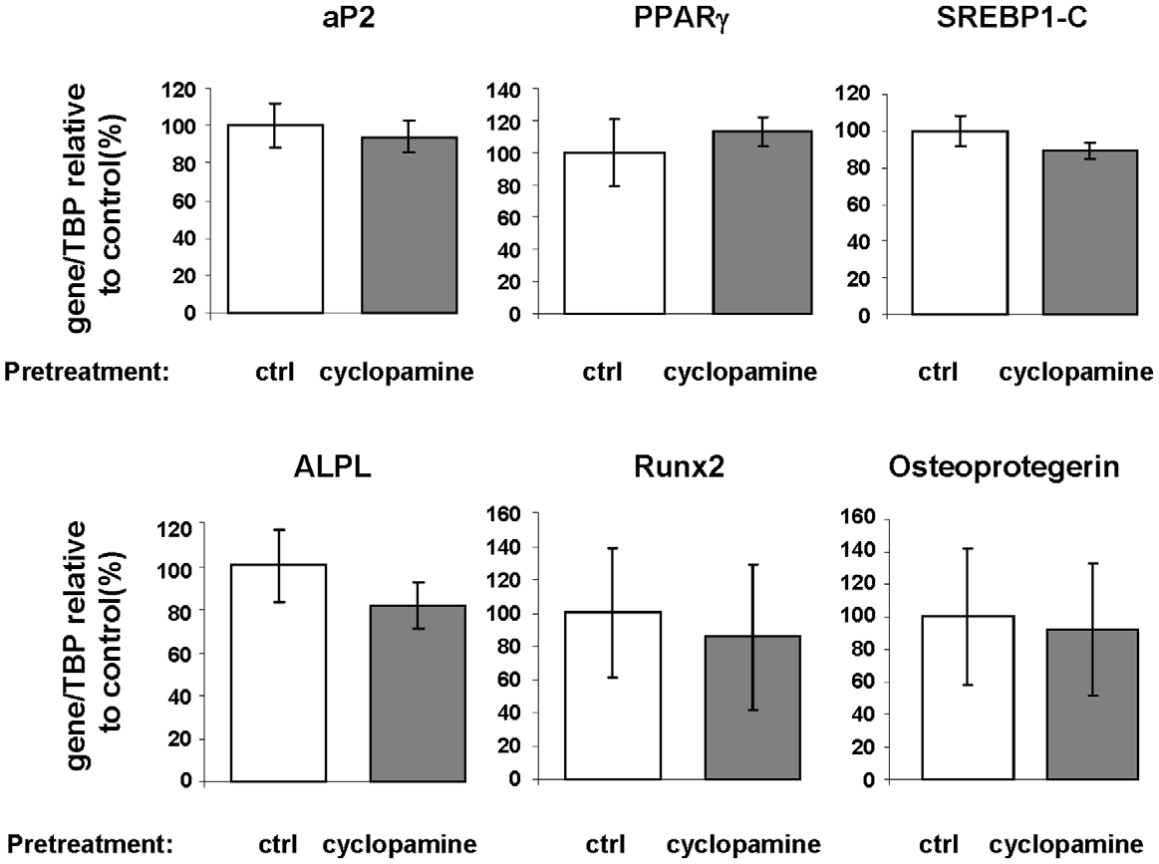
Cell-arrest induced by inhibition of Hh signaling does not affect hMSC differentiation. hMADS cells were synchronised as described in “Methods” and treated with 5 µM cyclopamine. After 24 hours cyclopamine was removed and the subconfluent cells were treated with an adipocyte or an osteoblast differentiation cocktail. After 10 days mRNA were extracted and aP2, PPARγ, SREBP1-c, Runx-2, osteoprotegerin and ALPL expressions were evaluated by quantitative RT-PCR. Results are representative of three independent experiments.

Cell-cycle arrest is though to be necessary for cell differentiation of hMSC. We decided to test whether the accumulation of cells in G0/G1 phase induced by Hh signaling inhibition could favor cell differentiation. Cells were plated at low density and synchronized as described above. Cells were then treated with cyclopamine. After 24 hours cyclopamine was removed and the subconfluent cells were incubated in an adipocyte or an osteoblast differentiation medium. After 10 days RNA were extracted and differentiation was evaluated through the expression of markers. (Figure 8). When differentiation was induce before the cells had reached confluence, cells were less differentiated than under “classical conditions” and C/EBP-α was expressed at low level, that did not allowed quantification. So, instead of C/EBP-α we analyzed the expression of another adipocyte marker: SREBP1-c. No significant difference was observed between conditions.

## Discussion

MSC are used extensively to unravel molecular processes leading to cell differentiation. These cells are also currently used in several cellular therapy clinical trials [28]. Thus, a precise understanding of the self renewal properties of these cells, i.e. their ability to proliferate without losing their differentiation ability, seems of particular importance. Several molecules control hMSC self-renewal such as FGF-2 [3], Activin A [4] and Wnt [5]. Here, we show that Hh signaling regulates proliferation and clonogenicity of hMSC. Hh is known to control self renewal of various stem cell types during embryogenesis [10], [11], [12], [15], [29]. It has also important functions in adult stem cells such as stem cells of the hair follicle epithelium [13], of the gastrointestinal tract [30], in the sebaceous lineage [31] or in the particular case of cancer stem cells [9], [15],[16]. hMSCs appear as a new adult stem cell type regulated by Hh. The mechanism appears somehow unusual since, in contrast to what is observed in other non-cancer stem cells, hMSC are endowed with a basal level of Hh signaling. This basal activity appears necessary and sufficient to maintain hMSC proliferation since proliferation is not further stimulated by Hh treatment of the cells [17]. What maintains this basal Hh signaling activity? In hMADS cells, Ihh and Dhh mRNA are detectable by real time PCR, but at low levels [17], [18]. It is unclear whether this low level of messenger is sufficient to produce enough ligand to maintain a basal activity of Hh signaling. TGF has been shown to induce Hh signaling under some circumstances [32], [33]. However, Hh and TGF pathways are thought to interact downstream of Smo. Since cyclopamine targets Smo it should not affect an induction of Hh signaling by TGF. Finally, it could be envisioned that the basal level of Hh signaling is ligand-independent and is maintained by an incomplete inhibition of Smoothened by Patched. Indeed, although the precise mechanism that leads Ptc to inhibit Smo is not resolved, it is well established that a decrease in Ptc expression, or activity, is associated with an increase in Hh signaling. This is observed in Gorlin syndrome which is associated with Ptc mutations [15]. Such an uncoupling between Ptc and Smo in hMSC could be envisioned in hMSC.

In several stem cells and cancer cells Hh acts as a survival factor [25], [26], [27], [34]. Inhibition of Hh signaling in hMSC did not modify their viability, indicating that the cell cycle was affected. Hh has been reported to control the proliferation of several cell types through various molecular mechanisms such as; an increase in cyclin D expression [15], [16], modulation of pRb phosphorylation through cyclin D and cyclin E in cerebellar granule neuron precursors [35], the increase in E2F1, CDC2 and CDC45L in human keratinocytes overexpressing Gli-2 [36] or, an increase in the association of the tumor suppressor p53 with mdm2 in cells expressing Hh or a constitutively active smoothened [37]. In hMSC, our experiments indicate that cyclopamine delays the cells in the G0/G1 phase. This is associated with an increase in the active form of pRB that can be linked to the associated decrease in cyclin A. Indeed, cyclin A binds cdk2 that phosphorylates and inactivates pRb during the late G1 and S phases. Such a decrease in cyclin A expression by cyclopamine has previously been documented in ovarian carcinoma [38]. In addition, cyclopamine decreases the phosphorylation of MAP kinases which are well-described activators of cell proliferation. Interactions between Hh and ERK signaling pathway have previously been observed in cancer cells such as melanoma [34]. Thus, in hMSC basal Hh activity appears necessary for cyclin A expression, pRb phosphorylation and ERK activity.

Interestingly, although Hh signaling inhibition decreases hMSC proliferation and clonogenicity it does not affect their differentiation potential. Likewise, the cell-arrest state induced by inhibition of Hh signaling appears insufficient to favor a differentiation mechanism. This is somehow different to what has been observed with FGF-2 [3]. Indeed, hMSC that are deprived of FGF-2 during proliferation have lower adipogenic potential, even when FGF-2 is removed during differentiation. Thus, Hh signaling controls the proliferation rate of hMSC but does not hinder their further ability to differentiate. By contrast, Hh signaling is known to interfere with hMSC differentiation [17], [18]. Our results suggest that Hh signaling function as a morphogen that, at basal level, is necessary for optimal hMSC proliferation and clonogenicity, while it affects cell differentiation when activated.

Together, this study reveals that endogenous Hh signaling controls the proliferation and clonogenicity of hMSC. This new function for Hh signaling, together with previous reports demonstrating that Hh controls hMSC differentiation, highlight the cardinal function of this morphogen in hMSC homeostasis.

## Materials and Methods

### Cell cultures

For the isolation of hMADS-2 and hMADS-3 cells from young donors, adipose tissues were obtained with the written consent of the parents as surgical scraps from surgical specimen of various surgeries, as approved by the Centre Hospitalier Universitaire de Nice Review Board, as previously described [22], [39]. To follow French bioethics laws, donors remain anonymous and files are kept at the Centre Hospitalier Universitaire de Nice. Self-renewal properties and ability of these cells to differentiate into adipocytes and osteoblasts have been extensively described in [3], [39], [40]. hMSC isolated from bone marrow were purchased from Cambrex (Cambrex, Emerainville, France). Cells were grown in Dulbecco's modified Eagle's medium (DMEM) supplemented with 10% fetal calf serum (FCS), 2,5 ng/ml human fibroblast growth factor (hFGF)-2 (for hMADS cells only), 60 µg/ml penicillin, and 50 µg/ml streptomycin. hFGF-2 was removed when cells reached confluence.

### hMADS cell differentiation

Adipocyte differentiation was performed as described previously [39]. Confluent cells were cultured in DMEM/Ham's F12 media supplemented with transferrin (10 µg/ml), insulin (0.86 µM), triiodothyronine (0.2 nM), dexamethasone (1 µM), isobutyl-methylxanthine (100 µM) and rosiglitazone (500 nM). Three days later, the medium was changed (dexamethasone and isobutylmethylxanthine were omitted). For osteoblasts, confluent cells were cultured in α-MEM medium containing 1% FCS supplemented with 200 µM L-ascorbic acid phosphate, 10 mM α-glycerophosphate, 100 nM dexamethasone, and 10 ng/ml epidermal growth factor. For simultaneous adipocyte and osteoblast differentiation, confluent cells were cultured in the differentiation medium consisting of 50% adipogenic and 50% osteogenic media.

### Cell proliferation assays

Cells were plated onto 6-well plates (4,500 cells per cm^2^). When indicated, cyclopamine was added 24 hours after cell plating in order to avoid a potential effect of the compounds on cell attachment. At the appropriate time, adherent cells were dissociated in 0.25% trypsin/EDTA and counted with a Coulter counter. For each experiment, three wells per condition were counted.

### Apoptosis assays

Apoptosis was assessed by measuring membrane redistribution of phosphatidylserine using an annexin V-FITC apoptosis detection kit (Invitrogen, Cergy Pontoise, France). According to the furnisher protocol, after cyclopamine treatment, cells were collected, washed with PBS and resuspended in 500 µL of staining solution containing FITC-conjugated annexin V antibody and propidium iodide (PI). After incubation at room temperature for 20 min, cells were analysed by flow cytometry. Basal apoptosis and necrosis were identically determined on untreated cells. Staurosporine was used as a positive control. Analysis was performed on a Calibure FACS (BD, Le Pont de Claix, France) and at least 10,000 events were acquired per sample.

### Cell cycle analysis by FACS

hMADS cells were synchronised before cell cycle assays. Briefly, cells were plated on 75-mm dishes (4,500 cells/cm^2^) and grown in DMEM 10% FCS and hFGF-2. After 24 hours the serum was removed for 48 hours to arrest cells in G0/G1. Then, hMADS cells were incubated in growth medium (10% FCS and hFGF-2) for 24 h in presence or in absence of cyclopamine (5 µM). Cells were trypsinized, fixed in cold 70% ethanol and then permeabilized in a PBS/1% triton (v/v) solution for 10 minutes. Cell cycle distribution was evaluated by cyclin A and PI double labelling. Cells were incubated with an anti-cyclin A antibody (Novocastra, Rungis, France) for one hour followed by an incubation with an Alexa Fluor 488 conjugated-antibody (Invitrogen) for 30 minutes. Cells were then resuspended in 500 µl PBS containing NP40 (0,1%), RNase A (20 µg/ml) and PI (40 µg/ml). Analysis was performed on a Calibure FACS (BD Biosciences) and at least 10,000 events were acquired per sample.

### Cell transfection

hMADS cells were transfected with a siRNA directed against Gli2 (seq: GAU-CUG-GAC-AGG-GAU-GAC-UTT) or a non-relevant siRNA with HiPerfect Transfection Reagent (Qiagen, Courtaboeuf, France) as recommended by the manufacturer. Briefly, cells were seeded at high density (30,000 cells/cm^2^) in 6 or 12 well plates. After 24 hours cells were transfected with 50 nM siRNA previously mix with HiPerfect. For proliferation assays, cells were seeded in 12-well plates and counted 3 days after transfection. For clonal assays cells were transfected in 6-well plates, trypsinized 24 hours later and seeded at 10 cells/cm^2^ in 75-mm dishes.

### Clonal assays

Cells were plated at a density of 10 cells/cm2 in 75-mm dishes. When indicated, cyclopamine was added 24 hours after cell plating. 21 days after plating cells were fixed with 0.25% glutaraldehyde and stained with 0.1% crystal violet. Colonies containing at least 40 cells were enumerated under a light microscope. Medium was changed 3 times a week.

### Preparation of Cell Extracts and Western Blot Analysis

Cells were lysed in stop buffer (50 mM Hepes, pH 7.2, 150 mM NaCl, 10 mM ethylene-diamine-tetra-acetate, 10 mM Na4P2O7, 2 mM Na3VO4, 100 mM NaF, 1% Triton X-100 (v/v) in presence of Complete TM inhibitor cocktail (Roche Molecular Biochemicals, Paris, France). Primary antibodies were pRb (BD Bioscience), cyclins A, E1 and D1 (SantaCruz, CA) pMAPK, total MAPK (Cell signaling technologies) and tubulin (Sigma). Secondary horseradish peroxidase-conjugated antibodies were purchased from Dako (Trappes, France) or Promega. Western Blots were revealed using a ChemiDoc XRS System from Bio-Rad. For quantification, Western Blots from at least three independent experiments were analysed using “Quantity One” software from Bio-Rad (version 4.6.8). The intensity of the signal in the control condition was taken as 100%. Statistically differences between conditions were analyzed using unpaired Student's t test performed using Micrococal Origin version 6.0.

### RNA extraction and analysis

Total RNA were extracted with the TRI-Reagent kit (Souffelweyersheim, France) according to manufacturer's instructions. Total RNA was subjected to real-time quantitative reverse transcription (RT)-polymerase chain reaction (PCR) analysis as described in [17]. Primers were designed using Primer Express software (Applied Biosystems, Courtaboeuf, France) and validated by testing PCR efficiency using standard curves (85%≤ efficiency ≤115%). Gene expression was quantified using the comparative C_T_ (threshold cycle) method; TBP was used as reference. The list of the primers used is provided in supplemental Table S1.

### Statistical analysis

Data are shown as means ± SD. Statistically differences between groups were analyzed using unpaired Student's t test and are indicated on figures as follow: *p≤0.05, **p≤0.01, ***p≤0.001. Statistical analysis were performed using Micrococal Origin version 6.0 (Northampton, Ma).

## Supporting Information

Table S1 List of the primers used. The indicated primers were used for real-time quantitative reverse transcription (RT)-polymerase chain reaction (PCR) analysis.
(TIF)

## References

[1] Rosen CJ, Bouxsein ML (2006). Mechanisms of disease: is osteoporosis the obesity of bone?. Nat Clin Pract Rheumatol.

[2] Gimble JM, Zvonic S, Floyd ZE, Kassem M, Nuttall ME (2006). Playing with bone and fat.. J Cell Biochem.

[3] Zaragosi LE, Ailhaud G, Dani C (2006). Autocrine fibroblast growth factor 2 signaling is critical for self-renewal of human multipotent adipose-derived stem cells.. Stem Cells.

[4] Zaragosi LE, Wdziekonski B, Villageois P, Keophiphath M, Maumus M (2010). Activin A Plays a Critical Role in Proliferation and Differentiation of Human Adipose Progenitors.. Diabetes.

[5] Boland GM, Perkins G, Hall DJ, Tuan RS (2004). Wnt 3a promotes proliferation and suppresses osteogenic differentiation of adult human mesenchymal stem cells.. J Cell Biochem.

[6] Ingham PW, McMahon AP (2001). Hedgehog signaling in animal development: paradigms and principles.. Genes Dev.

[7] Hooper JE, Scott MP (2005). Communicating with Hedgehogs.. Nat Rev Mol Cell Biol.

[8] Varjosalo M, Taipale J (2008). Hedgehog: functions and mechanisms.. Genes Dev.

[9] Ruiz i Altaba A, Mas C, Stecca B (2007). The Gli code: an information nexus regulating cell fate, stemness and cancer.. Trends Cell Biol.

[10] Lai K, Kaspar BK, Gage FH, Schaffer DV (2003). Sonic hedgehog regulates adult neural progenitor proliferation in vitro and in vivo.. Nat Neurosci.

[11] Machold R, Hayashi S, Rutlin M, Muzumdar MD, Nery S (2003). Sonic hedgehog is required for progenitor cell maintenance in telencephalic stem cell niches.. Neuron.

[12] Molofsky AV, Pardal R, Morrison SJ (2004). Diverse mechanisms regulate stem cell self-renewal.. Curr Opin Cell Biol.

[13] Mill P, Mo R, Fu H, Grachtchouk M, Kim PC (2003). Sonic hedgehog-dependent activation of Gli2 is essential for embryonic hair follicle development.. Genes Dev.

[14] Li N, Singh S, Cherukuri P, Li H, Yuan Z (2008). Reciprocal intraepithelial interactions between TP63 and hedgehog signaling regulate quiescence and activation of progenitor elaboration by mammary stem cells.. Stem Cells.

[15] Teglund S, Toftgard R (2010). Hedgehog beyond medulloblastoma and basal cell carcinoma.. Biochim Biophys Acta.

[16] Scales SJ, de Sauvage FJ (2009). Mechanisms of Hedgehog pathway activation in cancer and implications for therapy.. Trends Pharmacol Sci.

[17] Fontaine C, Cousin W, Plaisant M, Dani C, Peraldi P (2008). Hedgehog signaling alters adipocyte maturation of human mesenchymal stem cells.. Stem Cells.

[18] Plaisant M, Fontaine C, Cousin W, Rochet N, Dani C (2009). Activation of hedgehog signaling inhibits osteoblast differentiation of human mesenchymal stem cells.. Stem Cells.

[19] Eggenschwiler JT, Anderson KV (2007). Cilia and developmental signaling.. Annu Rev Cell Dev Biol.

[20] Michaud EJ, Yoder BK (2006). The primary cilium in cell signaling and cancer.. Cancer Res.

[21] Riobo NA, Manning DR (2007). Pathways of signal transduction employed by vertebrate Hedgehogs.. Biochem J.

[22] Rodriguez AM, Pisani D, Dechesne CA, Turc-Carel C, Kurzenne JY (2005). Transplantation of a multipotent cell population from human adipose tissue induces dystrophin expression in the immunocompetent mdx mouse.. J Exp Med.

[23] Taipale J, Chen JK, Cooper MK, Wang B, Mann RK (2000). Effects of oncogenic mutations in Smoothened and Patched can be reversed by cyclopamine.. Nature.

[24] Yauch RL, Gould SE, Scales SJ, Tang T, Tian H (2008). A paracrine requirement for hedgehog signalling in cancer.. Nature.

[25] Clement V, Sanchez P, de Tribolet N, Radovanovic I, Ruiz i Altaba A (2007). HEDGEHOG-GLI1 signaling regulates human glioma growth, cancer stem cell self-renewal, and tumorigenicity.. Curr Biol.

[26] Cai C, Thorne J, Grabel L (2008). Hedgehog serves as a mitogen and survival factor during embryonic stem cell neurogenesis.. Stem Cells.

[27] Nolan-Stevaux O, Lau J, Truitt ML, Chu GC, Hebrok M (2009). GLI1 is regulated through Smoothened-independent mechanisms in neoplastic pancreatic ducts and mediates PDAC cell survival and transformation.. Genes Dev.

[28] Mizuno H (2009). Adipose-derived stem cells for tissue repair and regeneration: ten years of research and a literature review.. J Nippon Med Sch.

[29] Mao J, Kim BM, Rajurkar M, Shivdasani RA, McMahon AP (2010). Hedgehog signaling controls mesenchymal growth in the developing mammalian digestive tract.. Development.

[30] van den Brink GR (2007). Hedgehog signaling in development and homeostasis of the gastrointestinal tract.. Physiol Rev.

[31] Niemann C, Unden AB, Lyle S, Zouboulis Ch C, Toftgard R (2003). Indian hedgehog and beta-catenin signaling: role in the sebaceous lineage of normal and neoplastic mammalian epidermis.. Proc Natl Acad Sci U S A.

[32] Dennler S, Andre J, Alexaki I, Li A, Magnaldo T (2007). Induction of sonic hedgehog mediators by transforming growth factor-beta: Smad3-dependent activation of Gli2 and Gli1 expression in vitro and in vivo.. Cancer Res.

[33] Dennler S, Andre J, Verrecchia F, Mauviel A (2009). Cloning of the human GLI2 Promoter: transcriptional activation by transforming growth factor-beta via SMAD3/beta-catenin cooperation.. J Biol Chem.

[34] Stecca B, Mas C, Clement V, Zbinden M, Correa R (2007). Melanomas require HEDGEHOG-GLI signaling regulated by interactions between GLI1 and the RAS-MEK/AKT pathways.. Proc Natl Acad Sci U S A.

[35] Kenney AM, Rowitch DH (2000). Sonic hedgehog promotes G(1) cyclin expression and sustained cell cycle progression in mammalian neuronal precursors.. Mol Cell Biol.

[36] Regl G, Kasper M, Schnidar H, Eichberger T, Neill GW (2004). The zinc-finger transcription factor GLI2 antagonizes contact inhibition and differentiation of human epidermal cells.. Oncogene.

[37] Abe Y, Oda-Sato E, Tobiume K, Kawauchi K, Taya Y (2008). Hedgehog signaling overrides p53-mediated tumor suppression by activating Mdm2.. Proc Natl Acad Sci U S A.

[38] Chen X, Horiuchi A, Kikuchi N, Osada R, Yoshida J (2007). Hedgehog signal pathway is activated in ovarian carcinomas, correlating with cell proliferation: it's inhibition leads to growth suppression and apoptosis.. Cancer Sci.

[39] Rodriguez AM, Elabd C, Delteil F, Astier J, Vernochet C (2004). Adipocyte differentiation of multipotent cells established from human adipose tissue.. Biochem Biophys Res Commun.

[40] Elabd C, Chiellini C, Massoudi A, Cochet O, Zaragosi LE (2007). Human adipose tissue-derived multipotent stem cells differentiate in vitro and in vivo into osteocyte-like cells.. Biochem Biophys Res Commun.

