# Supplementary figures and images for: Inhibition of Hedgehog Signaling Decreases Proliferation and Clonogenicity of Human Mesenchymal Stem Cells
Source: PLoS One. 2011 Feb 3;6(2):e16798. doi: 10.1371/journal.pone.0016798 (PMC3033417; doi:10.1371/journal.pone.0016798)

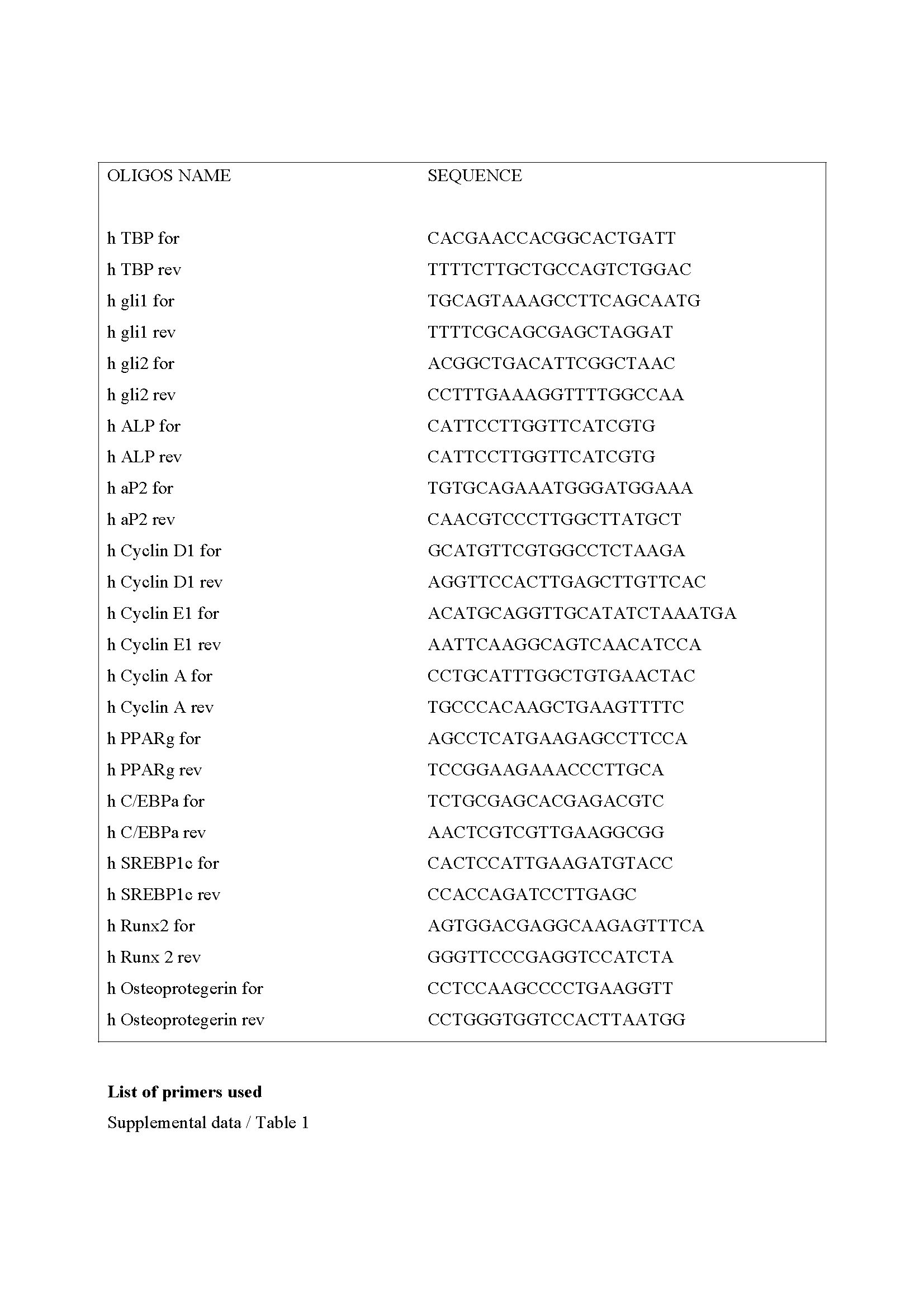

Supplement: Table S1 — List of the primers used. The indicated primers were used for real-time quantitative reverse transcription (RT)-polymerase chain reaction (PCR) analysis. (TIF) [file pone.0016798.s001.tif]
